# Multiplane Calcium Imaging Reveals Disrupted Development of Network Topology in Zebrafish *pcdh19* Mutants

**DOI:** 10.1523/ENEURO.0420-18.2019

**Published:** 2019-05-17

**Authors:** Sarah E.W. Light, James D. Jontes

**Affiliations:** Department of Neuroscience, Neuroscience Graduate Program, Ohio State University, Columbus, OH 43210

**Keywords:** calcium imaging, functional connectomics, pcdh19, zebrafish

## Abstract

Functional brain networks self-assemble during development, although the molecular basis of network assembly is poorly understood. Protocadherin-19 (*pcdh19*) is a homophilic cell adhesion molecule that is linked to neurodevelopmental disorders, and influences multiple cellular and developmental events in zebrafish. Although loss of *PCDH19* in humans and model organisms leads to functional deficits, the underlying network defects remain unknown. Here, we employ multiplane, resonant-scanning *in vivo* two-photon calcium imaging of developing zebrafish, and use graph theory to characterize the development of resting state functional networks in both wild-type and *pcdh19* mutant larvae. We find that the brain networks of *pcdh19* mutants display enhanced clustering and an altered developmental trajectory of network assembly. Our results show that functional imaging and network analysis in zebrafish larvae is an effective approach for characterizing the developmental impact of lesions in genes of clinical interest.

## Significance Statement

Non-clustered protocadherins are linked to neurodevelopmental disorders that include microcephaly, intellectual disability, autism spectrum disorders and epilepsy. In humans, mutations in protocadherin-19 (*PCDH19*) cause a female limited form of infantile epileptic encephalopathy and are associated with an increased incidence of schizophrenia and autism. In this study, we use large-scale calcium imaging to reveal that mutations in zebrafish *pcdh19* alter the development of brain network topology. This work is the first to use functional imaging to explore the effects of a clinically relevant mutation on brain-wide network assembly *in vivo*. We show that graph analysis of spontaneous network activity is a sensitive method for revealing subtle changes to network architecture in response to genetic perturbations.

## Introduction

Brain function and behavior are determined by the organization of the underlying neuronal networks, which are assembled through evolutionarily conserved developmental genetic programs. Thus, understanding the time course of network development can provide essential insights into both normal function and the effects of pathogenic mutations. The importance of proper neural development is clearest in some of the most prominent brain disorders, such as schizophrenia and autism spectrum disorder, which arise during the course of development and have been referred to as “connectopathies” (Friston and Frith, 1995; Geschwind and Levitt, 2007; Collin et al., 2016; Yamasaki et al., 2017). Many of the genes linked to these disorders are important for core neurodevelopmental processes, such as axon guidance, dendrite growth and synaptogenesis (Hussman et al., 2011; Lal et al., 2015). To better understand how structural and functional brain networks are constructed during development, it is necessary to visualize them during the course of their assembly. Not only will this inform our understanding of how network organization relates to brain function, but it can also provide essential insight into how genetic changes perturb brain function by altering developmental trajectories.

The zebrafish is an important vertebrate model system with several advantages for studying neural development. In addition to being well suited to both forward and reverse genetics, the small size and transparency of their embryos make them ideal for *in vivo* imaging of neural development (Jontes et al., 2000; Niell et al., 2004; Ahrens et al., 2013; Liu et al., 2018). With advances in imaging technology (Ahrens et al., 2013; Yang et al., 2016), data processing (Mukamel et al., 2009; Freeman et al., 2014; Pnevmatikakis et al., 2016) and the engineering of genetically-encoded calcium indicators (Chen et al., 2013), it is now possible to simultaneously image the activity of large numbers of neurons *in vivo* (Keller and Ahrens, 2015; Jercog et al., 2016). These advantages make it possible to follow both the structural and functional development of neural networks *in vivo* both during normal development and in lines harboring deleterious mutations.

The δ-protocadherins (δ-pcdhs) comprise a family of homophilic cell adhesion molecules that are differentially expressed in the developing nervous system (Kim et al., 2007; Blevins et al., 2011), and it has been proposed that this differential expression contributes to an adhesive code governing neural circuit organization (Wolverton and Lalande, 2001; Vanhalst et al., 2005; Light and Jontes, 2017). Several δ-pcdhs have been linked to epilepsy (Lal et al., 2015), autism (Marshall et al., 2008; Morrow et al., 2008; Butler et al., 2015), microcephaly (Aran et al., 2016), and intellectual disability (Kasnauskiene et al., 2012), suggesting that this family plays essential roles in vertebrate brain development. In humans, mutations in protocadherin-19 (*PCDH19*) cause a female-limited form of infantile epileptic encephalopathy (Dibbens et al., 2008; Depienne et al., 2009), with further work suggesting that *PCDH19* is also linked to a broader array of neural disorders, including autism (Piton et al., 2011). While loss of *pcdh19* affects visually-guided behaviors in zebrafish (Cooper et al., 2015) and heterozygous female mice show behavioral defects (Hayashi et al., 2017; Pederick et al., 2018), mutants in both models are homozygous viable and do not exhibit overt structural or anatomic phenotypes. Given the brain-wide expression of *pcdh19*, the viability of mutants, and the subtle behavioral defects, we wanted to investigate the influence of *pcdh19* on the overall development of network organization and activity.

Here, we used multiplane *in vivo* two-photon microscopy of zebrafish larvae expressing GCaMP6s (Ahrens et al., 2013; Chen et al., 2013) to visualize the development of network activity at the level of single neurons in wild-type larvae and in mutants lacking *pcdh19*. Using graph theory to analyze resting state functional networks (Bullmore and Sporns, 2009; Rubinov and Sporns, 2010), we show that *pcdh19* mutants display significant differences in several network measures as early as 3 d post-fertilization (dpf), as well as altered developmental trajectories of network properties. These data show that *in vivo* functional imaging in larvae harboring clinically relevant mutations can reveal quantitative changes during the development of functional networks.

## Materials and Methods

### Fish maintenance and transgenic lines

Adult zebrafish (*Danio rerio*) were maintained at ∼28.5°C and staged according to Westerfield (1995). All animal procedures were performed in accordance with the Ohio State University animal care committee’s regulations. Embryos were raised in E3 embryo medium (Westerfield, 1995) with 0.003% phenylthiourea (Sigma-Aldrich) to inhibit pigment formation.

The transgenic line *Tg(elavl3:GCaMP6s)* was established using the plasmid Tol2-elavl3-GCaMP6s, kindly provided by M. Ahrens (Janelia Farms, Addgene, plasmid #59531). This transgenic line was crossed with the *pcdh19* mutant line, *pcdh19^os51^* (Cooper et al., 2015). All experiments were performed in *pcdh19^-/-^* larvae.

### Imaging

Unanaesthetized larvae were embedded dorsal side up in 2% low melting point agarose, made up in E3 embryo medium. Imaging was performed on a custom-built resonant-scanning two-photon microscope. Briefly, excitation was provided by a Chameleon-XR Ti:Sapphire laser (Coherent, Inc.) tuned to 900 nm. We used a Nikon Apochromat 25×/1.1NA water-immersion objective for imaging. The resonant scanhead and controller, 3DMS robotic stage, GaAsP photomultiplier tubes and power supply were obtained from Sutter Instruments. A piezo-electric objective positioner (nPFocus250) was obtained from nPoint. Laser power was controlled by a Pockel’s cell (Conoptics Inc.). The microscope was run with ScanImage 5.2 (Vidrio Technologies). All other parts were obtained from Thorlabs. Image stacks of 12 optical sections (512 × 512) were collected at 1-s intervals for 15 min, with a pixel size of 1.3 μm. In older fish, there was a transient increase in fluorescence that accompanied the start of scanning (a stimulus artifact), so the first minute of imaging was removed before analysis for all movies.

For each group, we included data from eight to 11 larvae, which is in line with the number of fish used in comparable studies (Romano et al., 2015; Avitan et al., 2017). These were derived from at least two, separate crosses. The data presented here do not represent a longitudinal study, i.e., we did not image the same fish on successive days. Thus, the number of independent larvae imaged was 38 and 35 for wild-type and *pcdh19* mutants, respectively. Some movies showed drift in the z-direction over the first few minutes of imaging; these were excluded from the analysis presented here.

### Image segmentation and extraction of calcium signals

To extract ΔF/F traces from our calcium imaging movies, we used the constrained non-negative matrix factorization method (CNMF), as described in Pnevmatikakis et al. (2016), after the volumetric data were separated into timeseries for each imaged plane. Individual planes were cropped to exclude the tectal synaptic neuropil and autofluoresent skin, then processed using CNMF. For the CNMF pipeline, we used the following parameters: τ = 5, K = 300, merge_thr = 0.95. We overestimated the number of cells, as the program would discard cells during the refinement process. The processing pipeline was used as described at https://github.com/flatironinstitute/CaImAn-MATLAB. The data from each plane were then combined to produce a single dataset for an imaged larva.

### Network analysis

We used the Brain Connectivity Toolbox (https://sites.google.com/site/bctnet/) to calculate all network measures (Rubinov and Sporns, 2010). Correlation matrices were generated for each multiplane dataset, with entries being the pairwise Pearson’s correlation coefficient for the ΔF/F traces of neurons i and j. As the number of detected components varied among our datasets, the sizes of our corresponding networks also varied. To compare the distributions of edge weights, we generated a cumulative probability distribution for each dataset (50 bins between –1.0 and 1.0), which could then be averaged with other distributions within an experimental condition. to calculate complex network measures, we binarized our networks and calculated network measures across a series of global thresholds (0–0.7). The change in the distributions of edge weights between wild-type and *pcdh19* mutant networks meant that network densities differed for a given threshold. To calculate network measures, we used the undirected binary versions of each function. To normalize complex network measures, such as clustering coefficient, path length, small-worldness, and transitivity, we generated time-series randomized surrogate networks (Louie and Wilson, 2001; Zalesky et al., 2012) for each dataset. To convert our weighted correlation-based networks to unweighted networks, each graph was thresholded with the function threshold_absolute, then binarized using the weight_conversion function. Networks were thresholded across a range of values. The density of a network is the fraction of connections present divided by the total number of possible connections. The network density for each binarized network was calculated using density_und. Network densities within a group (developmental time and genotype) were averaged at a given threshold. The clustering coefficient represents the fraction of triangles around each node, as defined by the following equation:

$Cl=\frac{1}{N}\sum_{i\in N}\frac{2t_{i}}{k_{i}\left(k_{i}-1\right)}$. where $ti=\frac{1}{2}\sum_{j,h\in N}a_{ij}a_{ih}a_{jk}$ and $k_{i}$ represents the degree for node i.

We used clustering_coef_bu, which calculates a vector containing the clustering_coefficient for each node of a binary, undirected network. These values were averaged to provide a mean clustering coefficient for each network. We used the function charpath to calculate the characteristic path length, which is the mean shortest path between all node pairs in the network:

$L=\frac{1}{n}\sum_{i\in N}\frac{\sum_{j\in N,j\neq i}d_{ij}}{n-1}$, where $d_{ij}$ is the shortest path between nodes i and j.

To normalize both the clustering coefficient and the path length, we generated 100 time-series randomized surrogate networks (Louie and Wilson, 2001; Zalesky et al., 2012) for each experimental network, then calculated an average clustering coefficient and path length for each ensemble of randomized networks at each threshold. The thresholds of the randomized networks were adjusted to match the corresponding network densities of the experimental networks. The normalized clustering coefficient and path length were calculated as Cl*_norm_=* Cl/Cl*_rand_* and L*_norm_ =* L*/* L*_rand_*, respectively. Small-worldness was calculated as Cl*_norm_/* L*_norm_*. Network transitivity is related to the clustering coefficient and is defined as the ratio of observed triangles in the network to all possible triangles:

$$T=\frac{\sum_{i\in N}2t_{i}}{\sum_{i\in N}k_{i}\left(k_{i}-1\right)}$$

Transitivity is a scalar value generated by the function transitivity_bu for binary undirected networks. We normalized transitivity to the mean calculated from an ensemble of 100 time-series randomized networks, as was done for Cl and L. Assortativity is a correlation coefficient that measures the tendency of nodes to link to other nodes of similar degree:

$$r=\frac{l^{-1}\sum_{\left(i,j\right)\in L}k_{i}k_{j}-{\left[l^{-1}\sum_{\left(i,j\right)\in L}\frac{1}{2}\left(k_{i}+k_{j}\right)\right]}^{2}}{l^{-1}\sum_{\left(i,j\right)\in L}\frac{1}{2}\left(k_{i}{}^{2}+k_{j}{}^{2}\right)-{\left[l^{-1}\sum_{\left(i,j\right)\in L}\frac{1}{2}\left(k_{i}+k_{j}\right)\right]}^{2}}$$

Assortativity was calculated with the function assortativity_bin. In all cases (Cl*_norm_*, L*_norm_*, σ, T, and r), values for networks at each threshold were averaged within a group, and pairwise comparisons were made at each threshold between wild-type and *pcdh19* mutant groups for a given developmental time. Data were not analyzed blind, as data processing by CNMF was largely automated, and analysis was performed identically across all datasets with minimal user intervention. To determine the statistical significance of differences between wild-type and mutant networks, we used the unpaired, two-tailed Student’s *t* test for the normally distributed graph metrics, calculated at each threshold. For the comparisons of network measures across developmental time in Figure 3, we used ANOVA with Tukey HSD. The Kolmogorov–Smirnov test was used to determine the significance of changes in the distributions of correlation coefficients. Statistics were calculated either with RStudio or with Igor Pro (WaveMetrics).

## Results

### Multiplane calcium imaging in developing zebrafish larvae

**Figure 1.**
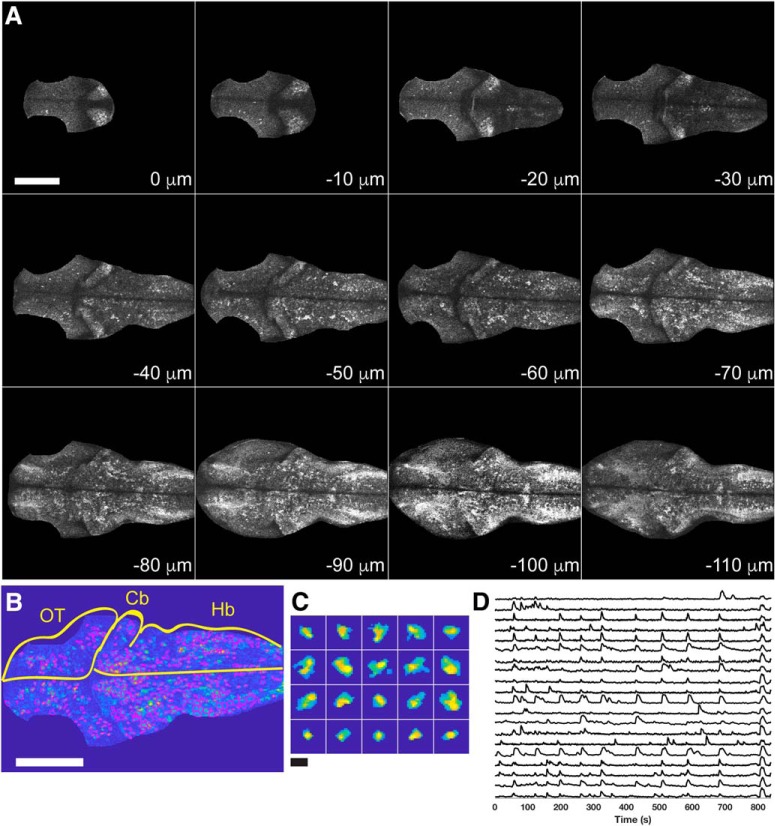
Two-photon imaging of GCaMP6s in zebrafish larvae. ***A***, Each panel shows an optical section from a single time point of a movie collected in a 4 dpf zebrafish larva. Sections are arranged from dorsal (0 μm) to ventral (–110 μm). The brain was cropped to remove skin fluorescence and signal from the synaptic neuropil of the optic tectum. Scale bar = 100 μm. ***B***, Detection of cells identified by constrained non-negative matrix factorization (Pnevmatikakis et al., 2016). ROIs outlined in magenta on top of an average image for a single imaging plane from a 4 dpf larva. Cb, cerebelleum; Hb, hindbrain; OT, optic tectum. Scale bar = 100 μm. ***C***, Selected ROIs. Scale bar = 15 μm. ***D***, Selected ΔF/F traces obtained from the segmented plane in ***B***, ***C***.

To investigate the development of functional brain networks in the zebrafish, we employed multiplane two-photon calcium imaging in transgenic zebrafish that expressed the genetically-encoded calcium indicator GCaMP6s under the control of the *elav3l* promoter, *Tg(elav3l:GCaMP6s)*. This promoter drives expression in nearly all neurons (Park et al., 2000; Ahrens et al., 2013). To provide a baseline for comparison to *pcdh19* mutants, we first visualized spontaneous neural activity in unanaesthetized wild-type larvae at 3, 4, 5, and 6 dpf that were immobilized in agarose. To sample neural activity patterns throughout the midbrain and hindbrain, we collected 12 optical sections spaced at 10 μm at 1-s intervals for 15 min (Fig. 1*A*; Movie 1). Constrained non-negative matrix factorization (Pnevmatikakis et al., 2016) was used to segment movies of each imaged plane (Fig. 1*B*,*C*) and to extract ΔF/F traces from identified spatial components [regions of interest (ROIs); Fig. 1*D*]. For each imaged larva, fluorescence traces from all imaged planes were combined into a single dataset. We analyzed 8–11 larvae per timepoint for a total of 38 wild-type datasets.

Movie 1. **GCaMP6s fluorescence in a wild-type 3 dpf zebrafish larva.** Shown is a movie assembled from a maximum intensity projection of all imaged planes in a wild-type 3 dpf larva. A Gaussian blur (radius = 0.5 pixels) was used here for display purposes but was not used for the analyzed data.
10.1523/ENEURO.0420-18.2019.video.1

### Development of zebrafish network organization

**Figure 2.**
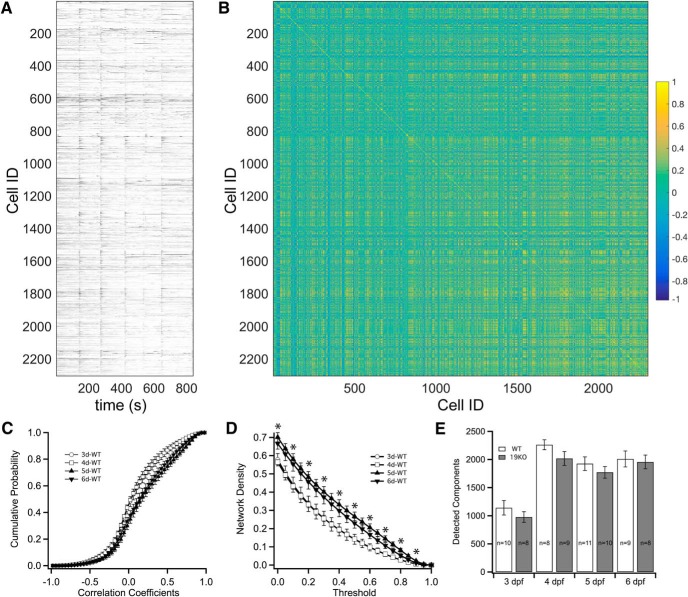
Development of zebrafish brain networks. ***A***, ΔF/F fluorescence traces for 2241 ROIs identified in a movie collected from a 4 dpf zebrafish larva. ***B***, A correlation matrix showing the pairwise correlation for all components in the network. ***C***, The average cumulative probability histograms for the correlation coefficients determined at each developmental time for wild-type larvae. Plots represent mean ± SEM. ***D***, The average network density as a function of threshold for edge weights. Plots represent mean ± SEM. Statistical significance was determined by ANOVA and a Tukey test. Exact *p* values are provided in the statistical table. ***E***, Shown are the numbers of segmented spatial components (ROIs) for wild-type and mutant larvae at each developmental time point. The numbers *n* are the number of larvae analyzed. The error bars represent SEM. *p < 0.05.

Individual datasets consisted of an array of fluorescence traces that represented a broad sampling of neural activity in the midbrain and hindbrain of zebrafish larvae (Fig. 2*A*). A prominent feature of these data was the presence of synchronous bursts of activity, which we observe as early as 3 dpf. This synchronous activity has been previously reported in zebrafish (Dunn et al., 2016; Avitan et al., 2017), and is similar to correlated activity that has been observed in other developing systems (Golshani et al., 2009). Using these datasets, we constructed correlation-based functional networks, with identified ROIs serving as nodes and pairwise correlation coefficients serving as edges (Fig. 2*B*). To begin to understand how these functional networks mature during development, we compared the distributions of correlation coefficients. As the number of detected cells and the sizes of corresponding networks varied among individual datasets, we generated cumulative probability distributions of edge weights for each network, allowing us to average them within an experimental group (Fig. 2*C*,*D*). The distribution of correlation coefficients was similar for 3 and 4 dpf and for 5 and 6 dpf, but showed a prominent shift between 4 and 5 dpf.

To explore the topology of network development, we applied graph theory to our datasets, as it provides a robust mathematical framework for analyzing functional brain networks and allows quantitative comparisons between experimental groups (Bullmore and Sporns, 2009; Rubinov and Sporns, 2010). A previous study characterized the development of neuronal cultures using multielectrode array recordings and graph theory, revealing the emergence of rich-club topology, the tendency of highly connected nodes to be linked to other highly connected nodes (Schroeter et al., 2015). More recently, graph measures were used to assess the influence of visual experience on the development of the zebrafish optic tectum (Avitan et al., 2017). To calculate network measures, we binarized each network across a range of thresholds (van Wijk et al., 2010). The network density is the ratio of connections in a network to all possible connections. At a given threshold, densities varied as a function of developmental age (Fig. 2*D*; Table 1), in accord with the time course of changes in the distribution of correlation coefficients (Fig. 2*C*). The number of ROIs detected increased between 3 and 4 dpf, but was stable between 4 and 6 dpf (Fig. 2*E*).

**Table 1.** Statistical Table

Figure	Test	*n*	Statistical significance
2*C*	Kolmogorov–Smirnov	3 dpf = 8,536,216; 5 dpf = 21,599,014	2.20E-16
		3 dpf = 8,536,216; 6 dpf = 26,218,604	2.20E-16
		4 dpf = 27,511,930; 5 dpf = 21,599,014	2.20E-16
		4 dpf = 27,511,930; 6 dpf = 26,218,604	2.20E-16
2*D*	One-way ANOVA + Tukey		
0		3 dpf = 10; 5 dpf = 11	0.00883753
		4 dpf = 8; 5 dpf = 11	0.00883753
0.1		3 dpf = 10; 5 dpf = 11	0.0257874
		4 dpf = 8; 5 dpf = 11	0.0236042
0.2		3 dpf = 10; 5 dpf = 11	0.0236042
		4 dpf = 8; 5 dpf = 11	0.0414124
0.3		3 dpf = 10; 5 dpf = 11	0.0145531
		4 dpf = 8; 5 dpf = 11	0.0308327
0.4		3 dpf = 10; 5 dpf = 11	0.00743578
		4 dpf = 8; 5 dpf = 11	0.0172431
0.5		3 dpf = 10; 5 dpf = 11	0.00333562
		4 dpf = 8; 5 dpf = 11	0.00801112
0.6		3 dpf = 10; 5 dpf = 11	0.000616054
		4 dpf = 8; 5 dpf = 11	0.00136372
0.7		3 dpf = 10; 5 dpf = 11	0.000408412
		4 dpf = 8; 5 dpf = 11	0.000880673
0.8		3 dpf = 10; 5 dpf = 11	0.000641302
		4 dpf = 8; 5 dpf = 11	0.00162331
0.9		3 dpf = 10; 5 dpf = 11	0.0104288
		4 dpf = 8; 5 dpf = 11	0.0195247
3*E*	One-way ANOVA + Tukey		
0.2		3 dpf = 10; 4 dpf = 8	0.0230188
0.5		3 dpf = 10; 5 dpf = 11	0.0497095
3*F*	One-way ANOVA + Tukey		
0.2		3 dpf = 10; 4 dpf = 8	0.0100918
0.3		3 dpf = 10; 4 dpf = 8	0.0460378
		3 dpf = 10; 5 dpf = 11	0.0475936
0.4		3 dpf = 10; 5 dpf = 11	0.0187628
0.5		3 dpf = 10; 5 dpf = 11	0.0226596
0.6		3 dpf = 10; 5 dpf = 11	0.0383578
4*A*	Kolmogorov–Smirnov	WT = 8,536,216; mut = 5,925,875	2.20E-16
4*D*		WT = 26,218,604; mut = 20,481,645	2.20E-16
4*H*	*t* test, unpaired, two-tailed		
0		WT = 9; mut = 8	0.0288994
0.4		WT = 9; mut = 8	0.0485647
0.5		WT = 9; mut = 8	0.0395246
0.6		WT = 9; mut = 8	0.0305472
0.7		WT = 9; mut = 8	0.0249533
0.8		WT = 9; mut = 8	0.0255265
0.9		WT = 9; mut = 8	0.0264769
			
5*A*		WT = 10; mut = 8	0.0338688
5*D*			
0.2		WT = 9; mut = 8	0.00163173
0.3		WT = 9; mut = 8	0.0148606
0.6		WT = 9; mut = 8	0.0244864
0.7		WT = 9; mut = 8	0.0118865
5*H*			
0.4		WT = 9; mut = 8	0.0467999
0.5		WT = 9; mut = 8	0.00795539
0.6		WT = 9; mut = 8	0.00800995
0.7		WT = 9; mut = 8	0.000432102
6*A*			
0.4		WT = 10; mut = 8	0.0392501
0.5		WT = 10; mut = 8	0.0304538
0.6		WT = 10; mut = 8	0.0136475
0.7		WT = 10; mut = 8	0.00459829
6*B*			
0.6		WT = 8; mut = 9	0.0400735
0.7		WT = 8; mut = 9	0.0336544
6*C*			
0.7		WT = 11; mut = 10	0.0418721
6*D*			
0.7		WT = 9; mut = 8	0.0366925
7*A*			
0.2		WT = 10; mut = 8	0.0251808
0.3		WT = 10; mut = 8	0.00195262
0.4		WT = 10; mut = 8	0.000275553
0.5		WT = 10; mut = 8	0.000550792
0.6		WT = 10; mut = 8	0.0111991
7*C*			
0		WT = 11; mut = 10	0.0357318

Summary of the statistics calculated in each figure, including *n*, *p* value, and type of test.

**Figure 3.**
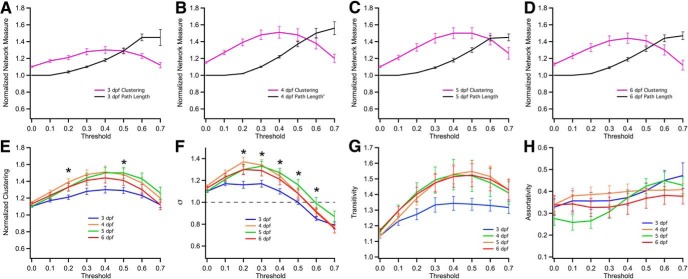
Developmental time course of network measures. ***A–D***, The average normalized clustering coefficients (pink) and path lengths (black) for networks as a function of threshold at 3 dpf (***A***), 4 dpf (***B***), 5 dpf (***C***), and 6 dpf (***D***). ***E***, The normalized clustering coefficient calculated as a function of edge weight threshold at each developmental time. ***F***, Small-worldness calculated as a function of edge weight threshold at each developmental time. Values above 1 (dotted-line) indicate the presence of small-world organization. ***G***, Transitivity calculated as a function of edge weight threshold at each developmental time. ***H***, Assortativity calculated as a function of edge weight threshold at each developmental time. No statistically significant differences were found using ANOVA and the Tukey test. Exact *p* values are provided in the statistical table. *p < 0.05.

We next calculated more complex network measures at each developmental time, across a range of thresholds. For example, the normalized clustering coefficient measures the proportion of closed triangles between a node and its neighbors (Watts and Strogatz, 1998). As correlation-based networks exhibit enhanced clustering due to the transitive nature of correlation coefficients (Zalesky et al., 2012), we normalized the clustering coefficient (Cl*_norm_*) for each network against the mean calculated from 100 corresponding, time-series randomized networks (Louie and Wilson, 2001; Zalesky et al., 2012), adjusting the thresholds of the randomized networks to match the network density of the experimental network (Fig. 3*A–D*). Similarly, we calculated normalized characteristic path lengths (L*_norm_*), which represents the average shortest path linking any two nodes in the network (Fig. 3*A–D*). While the path length remained relatively constant for the investigated time-points, the clustering coefficient increased between 3 and 4 dpf (Fig. 3*A*,*B*), but remained stable thereafter (Fig. 3*B–E*).

Nervous systems ranging from *C.elegans* to the human brain exhibit small-world properties, characterized by high clustering of connections and short path lengths (Sporns, 2010; Fornito et al., 2016). To explore this property in developing zebrafish brain networks, we calculated small-worldness (*σ*) as the ratio of Cl*_norm_/* L*_norm_*, with values above 1 taken as evidence for small-world organization (Watts and Strogatz, 1998). Zebrafish larvae showed evidence of small-worldness across a range of thresholds at all ages (Fig. 3*F*), with an evident enhancement between 3 and 4 dpf, driven primarily by changes in the clustering coefficient at high thresholds (Fig. 3*E*).

Transitivity is a network measure similar to the clustering coefficient, measuring the probability that if a node is connected to two other nodes, those nodes will also be connected. The normalized transitivity exhibited a similar developmental time course (Fig. 3*G*) to what was found for clustering (Fig. 3*E*), increasing between 3 and 4 dpf and remaining stable thereafter. We additionally calculated the degree assortativity, which is similar to rich-club organization and measures the tendency of nodes to connect to other nodes of similar degree: highly connected nodes will tend to be connected to other highly connected nodes and sparsely connected nodes will tend to be connected to other sparsely connected nodes. We observed positive assortativity across a range of thresholds at all ages, but found little evidence for developmental changes (Fig. 3*H*). Overall, our data show that the complex network measures are relatively stable between 4 and 6 dpf, suggesting that the basic scaffold of the brain is established early during development.

### Altered network development in *pcdh19* mutants

**Figure 4.**
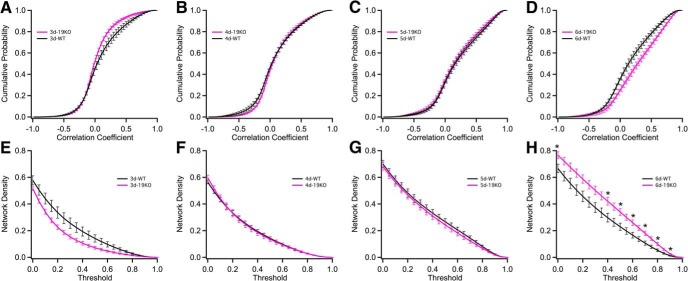
Development of basic network properties in wild-type and *pcdh19* mutant larvae. ***A–D***, The cumulative probability distributions for correlation coefficients of wild-type (black) and *pcdh19* mutant (pink) larvae at 3 dpf (***A***), 4 dpf (***B***), 5 dpf (***C***), and 6 dpf (***D***). Plots represent mean ± SEM. Statistical significance was computed using a Kolmogorov–Smirnov test. Exact *p* values are provided in the statistical table. ***E–H***, The network density as a function of correlation coefficient threshold for wild-type (black) and and *pcdh19* mutant (pink) networks at 3 dpf (***E***), 4 dpf (***F***), 5 dpf (***G***), and 6 dpf (***H***). Plots represent mean ± SEM. Significance was computed using an unpaired, two-tailed Student’s *t* test for each 0.1 increment. Exact *p* values are provided in the statistical table. *p < 0.05.

To determine how loss of *pcdh19* affects brain development in zebrafish larvae, we crossed the *Tg(elav3l:GCaMP6s)* fish with a previously published *pcdh19* mutant line, *pcdh19* and performed calcium imaging, as described above (Movie 2). In all, we imaged 8–10 larvae at each developmental time, for a total of 35 larvae imaged. Calcium imaging datasets from *pcdh19* mutants were qualitatively similar to those obtained from wild type: similar numbers of cells were identified, and these cells had comparable levels of activity (Fig. 2*E*). Like wild-type larvae, the *pcdh19* mutants exhibited a developmental increase in correlated activity (Fig. 4*A*). However, mutant networks show reduced correlation at 3 dpf, but enhanced correlation at 6 dpf (Fig. 4*A*,*D*), revealing that the developmental trajectory of functional connectivity is altered in *pcdh19* mutants. The change in the distribution of correlation coefficients was also reflected by changes in network density across a range of thresholds (Fig. 4*E–H*).

Movie 2. **GCaMP6s fluorescence in a pcdh19 mutant 3 dpf zebrafish larva.** Shown is a movie assembled from a maximum intensity projection of all imaged planes in a pcdh19 mutant 3 dpf larva. A Gaussian blur (radius = 0.5 pixels) was used here for display purposes but was not used for the analyzed data. 10.1523/ENEURO.0420-18.2019.video.2

**Figure 5.**
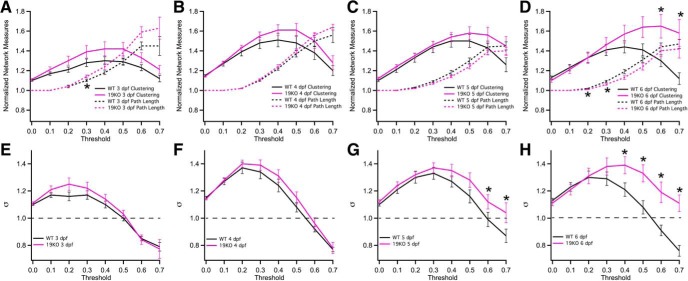
Altered development of small-worldness in *pcdh19* mutants. ***A–D***, Comparison of the normalized clustering coefficient (solid) and path length (dotted) between wild-type (black) and *pcdh19* mutant (pink) larvae at 3 dpf (***A***), 4 dpf (***B***), 5 dpf (***C***), and 6 dpf (***D***). Plots represent mean ± SEM. Significance was computed using an unpaired, two-tailed Student’s *t* test. Exact *p* values are provided in the statistical table. ***E–H***, Comparison of small-worldness between wild-type (black) and *pcdh19* mutant (pink) larvae at 3 dpf (***E***), 4 dpf (***F***), 5 dpf (***G***), and 6 dpf (***H***). Values above 1 (dotted-line) indicate the presence of small-world organization. Plots represent mean ± SEM. Significance was computed using an unpaired, two-tailed Student’s *t* test. Exact *p* values are provided in the statistical table, although these differences were not statistically significant. *p < 0.05.

**Figure 6.**
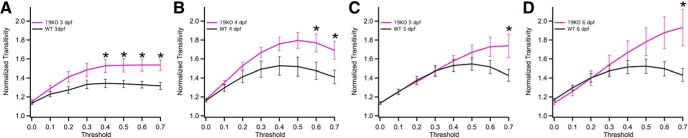
Developmental changes in transitivity in *pcdh19* mutants. ***A–D***, Comparison of transitivity between wild-type (black) and *pcdh19* mutant (pink) larvae at 3 dpf (***A***), 4 dpf (***B***), 5 dpf (***C***), and 6 dpf (***D***). Plots represent mean ± SEM. Significance was computed using an unpaired, two-tailed Student’s *t* test. Exact *p* values are provided in the statistical table. *p < 0.05.

**Figure 7.**
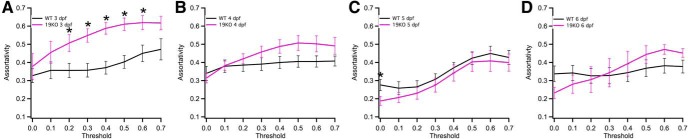
Development of assortativity in wild-type and *pcdh19* mutant larvae. Comparison of assortativity between wild-type (black) and *pcdh19* mutant (pink) larvae at 3 dpf (***A***), 4 dpf (***B***), 5 dpf (***C***), and 6 dpf (***D***). Plots represent mean ± SEM. Significance was computed using an unpaired, two-tailed Student’s *t* test. Exact *p* values are provided in the statistical table. *p < 0.05.

To determine the effects of *pcdh19* loss on network assembly, we compared complex network measures between mutant and wild-type larvae (Fig. 5). At 3 dpf, the *pcdh19* mutants displayed both higher clustering and a longer path length over a range of thresholds (Fig. 5*A*),

The slight enhancement of clustering in the mutants was maintained at 4 dpf (Fig. 5*B*) and 5 dpf (Fig. 5*C*), but became more pronounced at 6 dpf at the higher thresholds (Fig. 5*D*). Like wild-type networks, the mutant networks had small-world properties across a range of thresholds at all ages (Fig. 5*E–H*). At 3 dpf (Fig. 5*E*) and 4 dpf (Fig. 5*F*), there was no difference in small-worldness between wild-type and *pcdh19* mutant larvae. However, in contrast to wild type, *pcdh19* mutants showed a steady increase in small-worldness at 5 dpf (Fig. 5*G*) and 6 dpf (Fig. 5*H*). While normalized transitivity was largely stable in wild-type mutants between 4 and 6 dpf, *pcdh19* mutants showed an enhancement relative to wild type across all developmental times, as well as a steady increase between 3 and 6 dpf that was not observed in wild type (Fig. 6*A–D*). The increased transitivity at 6 dpf is consistent with the observed increase in clustering and small-worldness at 6 dpf. Degree assortativity was increased in *pcdh19* mutants at 3 dpf, compared to wild-type larvae (Fig. 7*A*). Subsequently, this difference was lost (Fig. 7*A–D*); degree assortativity was similar between wild-type and mutant larvae at 5 dpf (Fig. 7*C*) and 6 dpf (Fig. 7*D*). Collectively, our data show that loss of *pcdh19* dramatically alters the development of functional connectivity in zebrafish larvae. Differences in some network measures, such as transitivity and degree assortativity, are evident at 3 dpf, while differences in network clustering and small-worldness arise gradually during development. These results show that lesioning *pcdh19* alters networks organization, with an increased propensity for the clustering of neuronal connections.

### Spatial distribution of network changes

**Figure 8.**
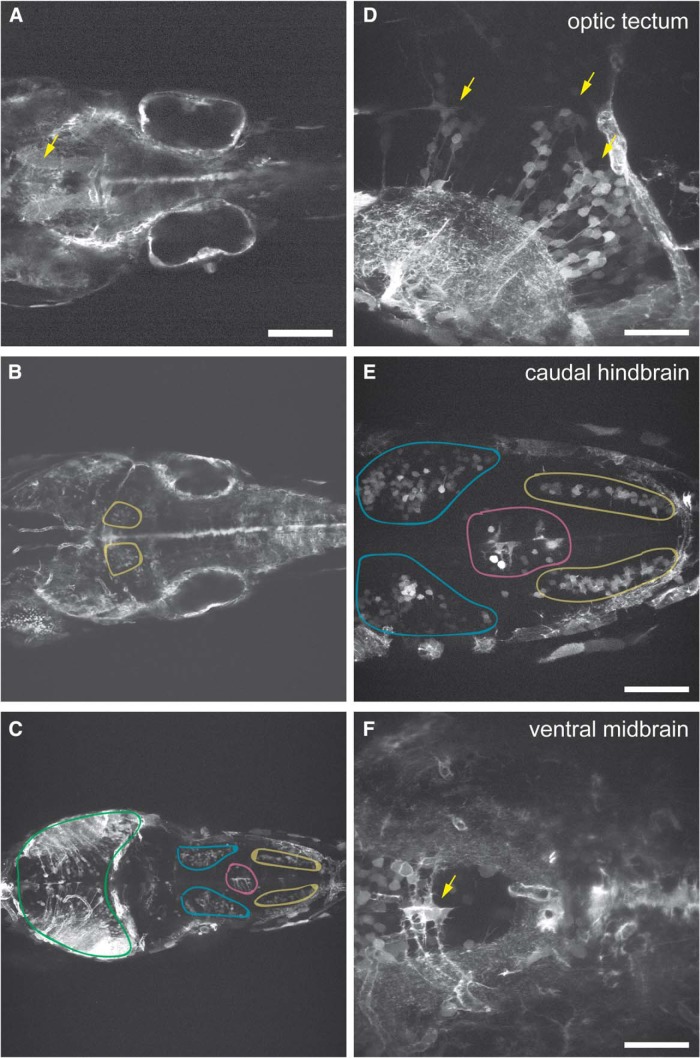
Expression of pcdh19 in BAC transgenic line. ***A–C***, Distribution of pcdh19 expression in the midbrain and hindbrain of *TgBAC(pcdh19:Gal4-VP16, 5xUAS:Lifeact-GFP)* larvae at 4 dpf. In addition to neurons and neural progenitor cells, pcdh19 is expressed in the neurovasculature and epithelia within the developing ear. ***A***, In the ventral brain, Lifeact-GFP labels both longitudinal (yellow arrow) and commissural axon bundles. ***B***, Expression is evident in a bilateral cluster of neurons in the anterior hindbrain, which likely correspond to the ARTR (yellow regions). ***C***, *pcdh19* is also present in limited clusters of neurons in the hindbrain (blue, magenta and yellow regions) and optic tectum (green region). ***D–F***, Higher magnification images show that within those regions that express *pcdh19*, it is present in only a fraction of neurons. ***D***, As shown previously, *pcdh19* is expressed in radial columns of neurons within the optic tectum (yellow arrows). ***E***, In addition to the ARTR, *pcdh19* labels bilateral clusters of neurons. ***F***, In the ventral midbrain, *pcdh19* labels small clusters of radial glia (yellow arrow), as well as neurons, axon tracts and vasculature. Scale bars = 125 μm (***A****–****C***), 50 μm (***D***, ***F***), and 63 μm (***E***).

We previously showed that *pcdh19* is expressed in distinct domains along the anterior neural tube during segmentation stages; expression is enriched in the anterior neural tube and forebrain, as well as stripes in the midbrain and hindbrain (Emond et al., 2009; Biswas et al., 2010). By 2 dpf, *pcdh19* is strongly expressed in the forebrain and hindbrain, as well as in cells lining the hindbrain and midbrain ventricles. We also showed that, later in development, *pcdh19* is expressed in columns of neurons in the optic tectum, generating a striped appearance (Cooper et al., 2015). Here, we further characterize a transgenic line, *TgBAC(pcdh19:Gal4-VP16, 5xUAS:Lifeact-GFP)*, that drives expression of the F-actin marker Lifeact-GFP in *pcdh19+* cells (Fig. 8). In the 5 dpf larval brain, *pcdh19* is expressed in the vasculature, as well as progenitor cells and neurons (Fig. 8). Expression is strongest in the ventral midbrain (Fig. 8*A*,*F*), the optic tectum (Fig. 8*C*,*D*), and the hindbrain (Fig. 8*B*,*C*,*E*). In each region, *pcdh19* is expressed in only a subpopulation of neural progenitor cells and neurons. For example, as we previously reported, *pcdh19* is present in radial stripes of tectal neurons, that are typically associated with one or more radial glia (Fig. 8*D*). Similarly, in the hindbrain, we observe *pcdh19* expression in subpopulations of neurons within bilateral clusters (Fig. 8*B*,*C*,*E*). Among these clusters, *pcdh19* is expressed in neurons that likely correspond to the anterior rhobencephalic turning region (ARTR; Dunn et al., 2016). In contrast to the broad expression of *pcdh19* in multiple brain regions, it does not appear to be expressed in the cerebellum.

**Figure 9.**
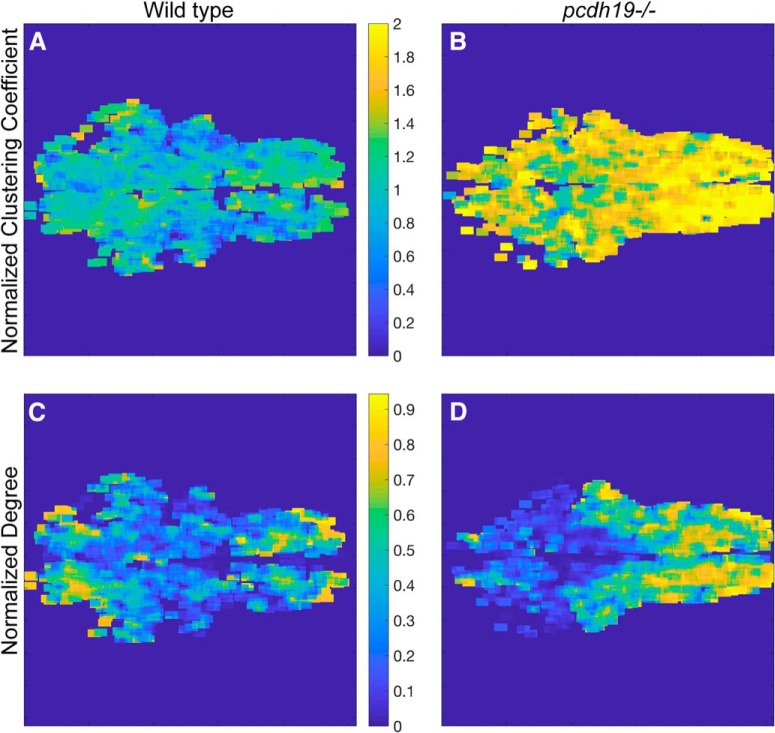
Spatial distribution of node-based measures in a 6 dpf larva. ***A***, ***B***, The normalized correlation coefficients were averaged in sliding 20 × 20 μm windows. Maps were generated from binarized networks that were thresholded at 0.7. The corresponding spatial maps show that there is little spatial organization in the distribution of clustering coefficients. Compared to wild-type larvae (***A***), there is a uniform elevation of the clustering coefficient throughout the brain in *pcdh19* mutants (***B***). ***C***, ***D***, The node degree for each network was normalized to the largest degree in that network. These normalized node degrees were averaged in sliding 20 × 20 μm windows. Wild-type larvae (***C***) consistently showed regions of high degree nodes in the anterior midbrain and in the hindbrain. In *pcdh19* mutants (***D***), there was a consistent spread of high degree nodes throughout the hindbrain.

To better understand the network alterations observed in *pcdh19* mutants, we investigated the spatial distributions of the normalized clustering coefficients and network degrees (Fig. 9). Both the clustering coefficient and degree are node-based metrics that can be mapped back on to the coordinates of the corresponding spatial components. To generate heat maps, we calculated the clustering coefficients and node degrees from binarized networks that were thresholded at 0.7, and averaged values for all spatial components within sliding 20 × 20 μm windows. In wild-type larvae, the normalized clustering coefficient (Fig. 9*A*) did not exhibit a marked spatial pattern. Although there were scattered “hotspots” in both the midbrain and hindbrain in some embryos, there was not a consistent pattern. In *pcdh19* mutants, we find here that the observed elevation in the normalized clustering coefficient is distributed across the brain and is not localized to a specific region (Fig. 9*B*), suggesting that the increase in clustering is distributed across the entire network. to compare the spatial distribution of degrees in networks of different sizes, we normalized each map to the highest degree in the individual network (values vary between 0 and 1). In contrast to the normalized clustering coefficient, the normalized node degree was less isotropic, with high-degree nodes consistently concentrated in the hindbrain and rostral midbrain of wild-type larvae (Fig. 9*C*). The *pcdh19* mutants exhibited a shift in the spatial distribution of high degree nodes, with a large expansion of area in the hindbrain (Fig. 9*D*). As *pcdh19* is expressed in clusters of neurons in the hindbrain (Fig. 8), this shift could be due to increased inter-connectivity of hindbrain neurons.

## Discussion

Non-clustered protocadherins are essential for nervous system development, as mutations in these genes have been linked to autism (Marshall et al., 2008; Morrow et al., 2008; Piton et al., 2011; Butler et al., 2015), schizophrenia (Gregório et al., 2009), intellectual disability (Kasnauskiene et al., 2012), microcephaly (Aran et al., 2016), and epilepsy (Dibbens et al., 2008; Lal et al., 2015). Our long-term objectives are to understand the cellular roles of these genes and how defects in these cellular roles lead to brain-wide defects during development. Pcdh19 is a δ2-pcdh and mutations in human *PCDH19* cause a female-limited form of early onset epileptic encephalopathy. Our goal here was to explore how loss of *pcdh19* influences brain network organization and development. We used *in vivo* multiplane two-photon calcium imaging to record neural activity from thousands of neurons in the midbrain and hindbrain regions of developing zebrafish, in both wild-type and *pcdh19* mutant larvae. To gain insight into the trajectory of network development, we collected data between 3 and 6 dpf, a period of dramatic growth and network maturation; by 6 dpf, zebrafish larvae already exhibit a rich array of behaviors (Orger and de Polavieja, 2017).

Neural activity in *pcdh19* mutants is qualitatively similar to that of wild-type larvae, as comparable numbers of active neurons are detected, which have similar levels of activity. Additionally, the brains of *pcdh19* mutant larvae do not display any overt morphogenetic or neuroanatomical defects. Yet, despite the fact that *pcdh19* is expressed in only a subpopulation of neurons, mutants exhibit several quantitative differences in network organization starting at 3 dpf, the earliest timepoint that we investigated. Both wild-type and mutant larvae show positive assortativity across all developmental times; *pcdh19* mutants show a significant elevation of assortativity at 3 dpf, but is indistinguishable from wild type at later times. At 3 dpf, *pcdh19* mutants have reduced levels of correlated activity and correspondingly lower network densities. This could reflect a delay or decrease in synaptogenesis at this early stage. By 6 dpf, the most prominent difference was the increase in network clustering in mutant larvae, as shown by increases in the clustering coefficient, small-worldness, and transitivity. In each case, the differences increase progressively over time, and are most pronounced at 6 dpf. These data suggest an aberrant patterning of synaptic connectivity in mutants lacking Pcdh19 function.

In functional MRI studies, resting state functional networks are routinely used to explore the topology of brain networks, relying on statistical correlations of activity patterns to identify functional connectivity between regions (Fornito et al., 2016). We have adopted this approach in the analysis of the larval zebrafish brain, as it allows the calculation of network properties for quantitative comparisons between genotypes and across development. Our calcium imaging data offered a number of obstacles to quantitative comparison between groups. While a powerful aspect of our approach is that our networks are assembled from calcium signals obtained largely from single neurons, the number of neurons detected and the size of the corresponding networks varied from one embryo to the next, restricting the network measures that could be averaged and compared across experimental groups. However, we observe robust quantitative differences in network properties between wild-type and *pcdh19* mutant larvae at 6 dpf. Moreover, we also see differences in the developmental trajectories of *pcdh19* mutants. Importantly, this is a relatively fast and efficient method to investigate changes in the development of network structure and dynamics, as quantitative differences can be observed even where structural defects are not apparent (no discernible change to synapse number or axon trajectories). Several groups have developed methods for registering transgenic and antibody-labeled larvae to model zebrafish brains (Randlett et al., 2015; Tabor et al., 2019). As markers for different brain regions and cell types become available, this will enable the assembly of cellular resolution atlases of the developing zebrafish brain. In future, as these atlases become better annotated, registration of our calcium imaging datasets will facilitate more detailed analyses and the construction of mesoscale functional networks.

The δ-pcdhs play diverse roles during development, having been implicated in nearly every aspect of brain assembly (Light and Jontes, 2017; Jontes, 2018). A major unresolved question for δ-pcdhs, as for other molecules, is the relationship of cellular phenotypes to changes in network-wide functional connectivity. Several studies showed that δ-pcdhs regulate cell movements and cell migration during development (Bradley et al., 1998; Aamar and Dawid, 2008; Emond et al., 2009; Biswas et al., 2010; Williams et al., 2018). Multiple lines of evidence suggest that δ-pcdhs are expressed in neural progenitor cells and regulate proliferation and neurogenesis (Zhang et al., 2014; Cooper et al., 2015; Fujitani et al., 2017). After neurogenesis, δ-pcdh family members have been implicated in axon outgrowth, guidance and arborization (Biswas et al., 2014; Hayashi et al., 2014; Leung et al., 2015). In addition, Pcdh11 has been shown to regulate dendritic branching in cultured cortical neurons (Wu et al., 2015). More broadly, *in vitro* studies show that δ-pcdhs can mediate cell sorting (Bisogni et al., 2018; Pederick et al., 2018). Defects in any of these developmental processes are likely to have an impact on patterns of synaptic connectivity and network topology. It has previously been shown that *pcdh19* mutants exhibit increased neurogenesis in the optic tectum, as well as disorganization in neuronal columns and patterns of arborization. We hypothesize that loss of *pcdh19* leads to network defects through the accumulation of several cellular perturbations. Loss of adhesion by Pcdh19 results in increased proliferation and differentiation of those neural progenitors that would normally have expressed *pcdh19*. Loss of contact-dependent axonal and dendritic growth could result in reduced fasciculation and inappropriate arborization, leading to promiscuous patterns of synaptogenesis. The increased number of neurons that form inappropriate synapses could lead to increased clustering in the mutant networks.

A host of genes have been linked to neurodevelopmental disorders through lineage studies and genome-wide association studies or whole genome sequencing approaches. Frequently, these genes participate in fundamental aspects of neural development (e.g., neurogenesis, neuronal migration or axon guidance), yet the mechanisms linking the genetic lesions to impaired brain function remain obscure. With the ease of making targeted genomic lesions with CRISPR/Cas9, the ability to screen large scale patterns of functional connectivity in the zebrafish brain and to follow the effects longitudinally during development offers a powerful approach to explore the biological mechanisms underlying complex brain disorders. Moreover, the zebrafish offers the possibility of combining these functional imaging approaches with simultaneous imaging and analyses of changes in axon tract development, synaptogenesis or neurogenesis. Thus, it should be possible to understand defects in functional networks in terms of the underlying changes in neural organization, and to link these changes to the cellular roles of altered genes.

## Synthesis

Reviewing Editor: Thomas McHugh, RIKEN Center for Brain Science Decisions are customarily a result of the Reviewing Editor and the peer reviewers coming together and discussing their recommendations until a consensus is reached. When revisions are invited, a fact-based synthesis statement explaining their decision and outlining what is needed to prepare a revision will be listed below. The following reviewer(s) agreed to reveal their identity: Eva Naumann.

The study uses state of the art two-photon microscopy, computational analysis tools (CaImAn) and the fast genetically encoded calcium sensor GCaMP6s to investigate how network activity as measured as fluorescence changes in automatically detected regions of interests changes over development from 3 to 6 days post fertilization (dpf). The analysis on wildtype fish reveals that network variables such as correlation and network density makes a significant transition between 3-4 dpf and later stages (5-6 dpf). The study goes on to showcase the power of functional imaging to between understand functional phenotypes of mutant models, particular those with subtle or undetectable anatomical/ cellular differences. This type of approach will also bridge understanding of phenotypes described at the synaptic or cellular connectivity levels. Pcdh19 is an interesting example, as little is known of roles in neuronal circuit formation and mutations are associated with a rare form of female X-linked epilepsy. The main conclusions of the paper are:

1) In normal brain development and during rest/spontaneous activity, several network measures increased (ie. network density and clustering patterns) from day 3 to day 4, but then plateau. From these results, they conclude that basic network organization of the zebrafish brain is established early, by day 4.

2) Some network features are already disrupted in Pcdh19 mutants by day 3 as they are less correlated, while aspects of network organization such as small worldness show increased tendency for clustering, especially distributed within the hindbrain.

Both referees find the study interesting and novel, with one referee commenting “This is a novel and worthwhile approach and they use the experimental power of the zebrafish to comprehensively describe the functional topography, which illuminates the general differences in activity pattern statistics” and the second adding, “Since the zebrafish is particularly well-suited for in vivo studies of neuronal networks dynamics in the developing CNS, this study will surely be of interest to many.”

After consulting with the referees and discussing their reviews we have reached a consensus. They have identified several main areas that would need to be addressed prior to reconsideration for publication.

They are as follows:

1. Statistical testing and reporting. Both referees find that currently reported tests (Student's -test) inappropriate for the analyses of these data, suggesting that ANOVAs with corrections for multiple comparisons and Kolmogorov-Smirnov tests would be better suited. One referee suggested the Avitan et al Current Biology paper cited in your manuscript could serve as a template for many of the issues they were concerned with, adding “I suggest the authors adopt similar statistics practices used for graph theory in that paper, include statistics for particular age groups and also use corrections when appropriate (e.g. for multiple comparisons they should use Bonferroni corrections). Also I would be curious to see how they calculated statistically significant level of co-activity of the neurons and how they excluded neuropil activation. Otherwise Kolmogorov-Smirnov would be far more appropriate in most of their figures.”

2. Both referees would like more data concerning the expression pattern of Pcdh19, given what they found to be inexact language in the manuscript. One referee commented “What is the expression pattern of Pcdh19 in the midbrain and hindbrain? The language in the paper is confusing (ie. brain-wide expression, subsets of neuron), and there is limited characterization in Cooper et al 2015. The relative expression of Pcdh19 in the midbrain versus the hindbrain should be described. This information would help gauge whether spatial differences in network density shown in Figure 8 could relate to higher prevalence of Pcdh19 expression in hindbrain.”, with second adding “It would be great if they had expanded on the broader view of what the specific mutation might actually do and the mention that it is only expressed in some cells is confusing do the authors know which cells?”

3. There was strong agreement between the referees concerning the lack of transparency and discussion of parameters and caveats of the analyses used. The points raised in our discussion included “Both the statistics and chosen variables are not explained in sufficient detail- and it is not clear how they ensured an unbiased recording of brain regions- did they use landmarks to search for the same imaging planes?”, with the reviewers also noting, “the analysis and result is based on the analysis of network variables calculated by software packages that is not well explained“.“ Some possible remedies would be to add supplemental figures that demonstrate the analysis processes on their data step by step and to make the data available in either raw form or with analyzed and aligned ROIs. A related comment was ”The authors commented on signal variability and low signal. Is this a feature of the model and its immature circuitry? Or are there considerations in the image processing, or in the choice of algorithms that may be more suitable to the model? Other zebrafish groups are now devising new computational tools for segmentation, processing, clustering analyses etc (Ahrens/Engert; Sumbre; Randlett pERK Z-brain atlas, etc). This discussion would be helpful for the field, as these approaches evolve.

4. An expansion of the discussion and clearer writing to explain the differences observed between mutant and wild-type fish in more detail and discuss the implications, with the referee commenting “little is known of the direct roles of Pcdh19 adhesion molecules in neuronal wiring, They could offer a few lines of speculation in the discussion, based on the human genetics and some molecular/genetic findings.“

Minor points:

1. Was the cytosolic version of the Elav::Gcamp line used? This should be specified. Was this a limitation for segmentation and signal:noise in this study? Other groups are now using the H2B-Gcamp line.
